# Genetic polymorphisms of MRPS30-DT and NINJ2 may influence lung cancer risk

**DOI:** 10.1515/med-2023-0655

**Published:** 2023-03-09

**Authors:** Shouchun Yan, Shouzhen Wu, Jia Wu, Qinlu Zhang, Yongjun He, Chao Jiang, Tianbo Jin

**Affiliations:** Department of Emergency Medicine, The Second Affiliated Hospital of Shaanxi University of Chinese Medicine, Xianyang 712000, Shaanxi Province, China; School of Nursing, Shaanxi University of Chinese Medicine, Xianyang 712046, Shaanxi Province, China; Department of Respiratory Medicine, The First Affiliated Hospital of Xi’an Jiaotong University, Xi’an 710061, Shaanxi Province, China; The Third Department of Neurology, The Second Affiliated Hospital of Xi’an Medical University, Xi’an 710038, Shaanxi Province, China; Key Laboratory of Molecular Mechanism and Intervention Research for Plateau Diseases of Tibet Autonomous Region, School of Medicine, Xizang Minzu University, No. 6, Wenhui East Road, Xianyang 712082, Shaanxi Province, China; Key Laboratory of Resource Biology and Biotechnology in Western China, Ministry of Education, School of Medicine, Northwest University, Xi’an 710069, Shaanxi Province, China; Key Laboratory of Molecular Mechanism and Intervention Research for Plateau Diseases of Tibet Autonomous Region, School of Medicine, Xizang Minzu University, Xianyang 712082, Shaanxi Province, China

**Keywords:** MRPS30-DT, NINJ2, lung cancer, single-nucleotide polymorphisms

## Abstract

Lung cancer is one of the malignant tumors, and genetic background is a risk factor in lung cancer that cannot be neglected. In this study, we aimed to find out the effect of MRPS30-DT and NINJ2 variants on lung cancer risk. In this study, the seven selected single-nucleotide polymorphisms (SNPs) of MRPS30-DT and NINJ2 were genotyped in 509 lung cancer patients and 501 healthy controls based on the Agena MassARRAY platform. Odds ratios and 95% confidence intervals were calculated by logistic regression analysis to evaluate association between gene polymorphisms and lung cancer risk. False-positive report probability was also used to assess false-positive results. Furthermore, the interaction between SNPs was analyzed by multifactor dimensionality reduction to predict lung cancer risk. We identified the genotype TA of rs16901963 (T < A) in MRPS30-DT as a protective factor against lung cancer, while rs16901963-TT was significantly associated with an increased risk of lung cancer. We also revealed that the effect of MRPS30-DT and NINJ2 variants on the risk of lung cancer was dependent on age, gender, smoking, and drinking status. In conclusion, this study first proved that MRPS30-DT and NINJ2 variants played important roles in affecting the susceptibility to lung cancer.

## 1 Introduction

Lung cancer is the most threatening malignancy worldwide, with the number of diagnosed cases increasing every year and the 5-year survival rate reaching 10% [1,2]. Risk factors, including smoking, biomass exposure, radiation, and air pollution, were considered to be significantly associated with an increased incidence of lung cancer [3]. However, studies on 10–25% of non-smoking lung cancer patients have demonstrated that internal gene mutations were correlated with abnormal regulation of lung cancer [4]. A large number of epidemiological studies have confirmed that genetic polymorphisms were key factors in the progression of lung cancer. Recently, genome-wide association studies have also identified various single-nucleotide polymorphisms (SNPs) in lung cancer susceptibility genes [5,6]. The discovery of these susceptibility loci, such as 3q28, 5p15.33, 13q12.12, 22q12.2, and 12q23.1, is a crucial step in revealing the genetic background of lung cancer in a specific population.

MRPS30-DT, also known as BRCAT54, is located on chromosome 5p12. It is a divergent transcript of MRPS30. In one article about breast cancer, Wu et al. [7] observed the overexpression of MRPS30-DT by microarray analysis. The higher the expression, the worse the prognosis of patients. Functional experiments have shown that knockdown of MRPS30-DT significantly inhibited the progression of breast cancer and promoted the apoptosis of breast cancer, indicating that MRPS30-DT may be an oncogene in breast cancer. On the contrary, Yang et al. have observed that overexpression of BRCAT54 significantly promoted the apoptosis of lung cancer cells [8]. Therefore, BRCAT54 may act as a tumor suppressor in non-small-cell lung carcinoma. The above results may be related to the heterogeneity of cancer. Furthermore, the genetic variants of MRPS30 region were found to be correlated with the risk of breast cancer [9,10]. However, the association between MRPS30 variants and lung cancer risk was not found. And the effect of polymorphisms in the divergent transcript (MRPS30-DT) on the risk of lung cancer has never been studied.

NINJ2 is located on chromosome 12p13.33 and encodes a transmembrane protein that mediated cell–cell and cell–extracellular matrix interactions during the development, differentiation, and regeneration of nervous system [11]. NINJ2 was observed to be overexpressed in colorectal cancer cells and can promote human colorectal cancer cell growth [12]. Moreover, its overexpression may accelerate the growth of glioma cells [13]. The effects of NINJ2 gene polymorphisms have been widely studied in stroke-related diseases and neurological disorders [14,15,16,17,18], while never in lung cancer. Choi et al. have applied array comparative genomic hybridization to human emphysema and identified NINJ2 with a higher fold change, thereby suggesting that NINJ2 was a potential gene involved in the pathogenesis of emphysema [19]. Thus, NINJ2 gene alterations may play an important role in lung disease, including lung cancer.

Therefore, we set up this case–control study and genotyped seven SNPs of candidate genes in patients with lung cancer and healthy controls. Our study first examined the potential influence of candidate genes on the risk of lung cancer, which provided a theoretical basis for deep understanding of the genetic background of lung cancer.

## 2 Materials and methods

### 2.1 Study population

This study enrolled 1,010 subjects from Shaanxi Cancer Hospital, including 509 lung cancer patients and 501 healthy controls. Lung cancer patients were all histopathologically diagnosed, and patients who underwent radiotherapy were not included in the case group. Patients with a family history of lung cancer, other cancers, other lung diseases, or immune diseases were excluded. During the same period, 501 healthy controls were recruited from the healthcare center and they had no history of cancer or chronic diseases. The information about clinical characteristics (age, gender, smoking and drinking status, etc.) of all subjects was collected from questionnaire.


**Ethical approval:** This study complied with Helsinki Declaration and was approved by theEthics Committee of the Second Affiliated Hospital of Shaanxi University of Chinese Medicine (201204). All participants were informed of the purpose and procedures of this study and signed the informed content.

### 2.2 SNP selection and genotyping

The SNPs of candidate were downloaded from the 1000 Genomes Project. Then, Haploview software was used to set parameters (Hardy–Weinberg equilibrium [HWE] > 0.01, minor allele frequency [MAF] > 0.05, call rate > 0.95 and *r* ^2^ < 0.80) to screen SNPs. In addition to ineffective and unspecific primers, MRPS30-DT (rs16901963 and rs2118763) and NINJ2 (rs118050317, rs75750647, rs7307242, rs10849390, and rs11610368) were screened out. The amplification primers and extension primers for these SNPs were designed through the Agena online platform, as displayed in Table A1. Genomic DNA of all subjects was isolated and extracted from the whole blood samples using the kit (Xi’an GoldMag Co. Ltd., Xi’an, China), and the spectrophotometer was used for the detection of genomic DNA concentration (NanoDrop2000, Thermo, MA, USA). SNP genotyping and data collection were done with the Agena MassARRAY platform and TYPER 4.0 (Agena Bioscience, CA, USA), respectively.

### 2.3 Statistical analysis

In this study, genotyping data were collated and processed in Excel, SPSS 18.0, and PLINK software for statistical analysis. *T*-test and chi-square test were used to analyze the differences in age, gender, and clinical indexes between the two groups, respectively. Chi-square test was utilized to determine whether the allele frequencies of SNPs in the control group conformed to HWE. Furthermore, the logistic regression analysis was introduced to evaluate the association between the genetic polymorphisms of candidate genes and the susceptibility to lung cancer under the allelic and genetic models, with the corresponding values of odds ratios (ORs) and 95% confidence intervals (CIs). After adjustment for age and gender, the corresponding OR and 95% CI were also calculated. False-positive report probability (FPRP) analysis was utilized to determine whether there were false positives in significant results. Besides, we carried out linkage disequilibrium (LD) analysis through Haploview 4.2 and used *D*′ to represent the LD degree of different loci on the same chromosome. We also conducted the logistic regression analysis to assess the correlation between haplotypes and lung cancer risk based on different stratified analyses. Multifactor dimensionality reduction (MDR) software package was utilized to predict the association between the selected SNPs and lung cancer risk. All *p*-values <0.05 indicated statistical significance.

## 3 Results

### 3.1 Demographic and clinical characteristics of subjects and SNP information

The clinical information of 509 patients with lung cancer (57.99 ± 10.56 years, 75% males and 25% females) and 501 healthy controls (60.29 ± 8.13 years, 71% males and 29% females) is summarized in Table 1. The statistical results showed that there were significant differences in age and smoking status between cases and controls (*p* < 0.001), while no significant differences in gender (*p* = 0.243) and drinking status (*p* = 0.096). In addition, we collected the clinical characteristics of participants, including lymph node metastasis, histological type, and clinical stage.

**Table 1:** Demographic and clinical characteristics of subjects

Variables	Case (*n* = 509)	Control (*n* = 501)	*p*-Value
Age (mean ± SD, years)	57.99 ± 10.56	60.29 ± 8.13	**<0.001** ^**a**^
≤59	266 (52%)	227 (45%)	
>59	243 (48%)	274 (55%)	
Gender			0.243^b^
Male	383 (75%)	354 (71%)	
Female	126 (25%)	147 (29%)	
Smoking status			**<0.001** ^**b**^
Yes	313 (62%)	116 (23%)	
No	141 (28%)	176 (35%)	
Alcohol consumption status			0.096^b^
Yes	146 (29%)	108 (22%)	
No	271 (53%)	153 (31%)	
Lymph node metastasis			
Yes	192 (38%)		
No	114 (23%)		
Histological type			
Adenocarcinoma	168 (33%)		
Squamous	164 (32%)		
Clinical stage			
I/II	82 (16%)		
III/IV	188 (37%)		

SD, standard deviation. ^a^ *p*-value was calculated by *t*-test. ^b^ *p*-value was calculated by chi-square test. Bold data means significant difference (*p* < 0.05).

The information about seven selected SNPs is shown in Table 2. The analysis results revealed that allele frequencies of seven SNPs were in line with HWE (*p* > 0.05). No significant differences in allele frequencies were found between the cases and controls.

**Table 2:** Information about selected SNPs in MRPS30-DT and NINJ2

Gene	SNP	Chromosome	Position	Allele A/B	Role	MAF		HWE *p*-Value	OR (95% CI)	*p*-Value
						Case	Control			
*MRPS30-DT*	rs16901963	5	44783000	T/A	Intron	0.437	0.428	0.715	1.04 (0.87–1.24)	0.674
*MRPS30-DT*	rs2118763	5	44787444	T/C	Intron	0.059	0.060	0.695	0.98 (0.68–1.42)	0.920
*NINJ2*	rs118050317	12	634980	C/G	Intron	0.108	0.105	0.635	1.03 (0.77–1.37)	0.844
*NINJ2*	rs75750647	12	638831	A/G	Intron	0.344	0.334	0.841	1.04 (0.87–1.25)	0.653
*NINJ2*	rs7307242	12	641529	A/T	Intron	0.126	0.132	0.695	0.95 (0.73–1.23)	0.700
*NINJ2*	rs10849390	12	646086	G/A	Intron	0.359	0.341	0.689	1.08 (0.90–1.30)	0.416
*NINJ2*	rs11610368	12	662624	A/G	Intron	0.116	0.124	0.838	0.93 (0.71–1.21)	0.576

SNP, Single-nucleotide polymorphism; MAF, minor allele frequency; HWE, Hardy–Weinberg Equilibrium; OR, odds ratio; 95% CI, 95% confidence interval.

### 3.2 Association of MRPS30-DT and NINJ2 polymorphisms with lung cancer risk

To evaluate the association between MRPS30-DT and NINJ2 polymorphisms and lung cancer risk, we performed the logistic regression analysis under different genetic models (co-dominant, dominant, recessive, and additive models) and the results are listed in Table 3. The results suggested that the heterozygote TA of rs16901963 in MRPS30-DT was significantly associated with a reduced risk of lung cancer (OR = 0.67, 95% CI: 0.50–0.89, *p* = 0.006) in contrast with wide genotype AA. However, the genotype rs16901963-TT was found to be related to an increased risk of lung cancer (OR = 1.55, 95% CI: 1.14–2.12, *p* = 0.005) under the recessive model. In Table 4, the results of FPRP indicated that rs16901963 was still associated with lung cancer risk (TA vs AA: power = 0.978, FPRP = 0.017, 0.050; TT vs TA-AA: power = 0.945, FPRP = 0.019, 0.055).

**Table 3:** Association of MRPS30-DT and NINJ2 genetic polymorphisms with lung cancer risk

**Gene**	**SNP**	**Model**	**Genotype**	**Case**	**Control**	**OR (95% CI)**	***p***-Value
*MRPS30-DT*	rs16901963	Co-dominant	TT vs AA	126	89	1.24 (0.88–1.76)	0.218
			TA vs AA	193	249	0.67 (0.50–0.89)	**0.006**
		Dominant	TT-TA vs AA	319	338	0.82 (0.63–1.07)	0.139
		Recessive	TT vs TA-AA	126	89	1.55 (1.14–2.12)	**0.005**
		Additive	—	—	—	1.05 (0.89–1.25)	0.544
*MRPS30-DT*	rs2118763	Co-dominant	TT vs CC	2	2	0.97 (0.13–7.47)	0.978
			TC vs CC	56	56	0.94 (0.63–1.40)	0.754
		Dominant	TT-TC vs CC	58	58	0.94 (0.64–1.39)	0.755
		Recessive	TT vs TC-CC	2	2	0.98 (0.13–7.51)	0.983
		Additive	—	—	—	0.94 (0.65–1.37)	0.763
*NINJ2*	rs118050317	Co-dominant	CC vs GG	9	4	2.02 (0.61–6.70)	0.248
			CG vs GG	91	97	0.90 (0.66–1.25)	0.535
		Dominant	CC-CG vs GG	100	101	0.95 (0.69–1.30)	0.742
		Recessive	CC vs CG-GG	9	4	2.06 (0.62–6.82)	0.235
		Additive	—	—	—	1.00 (0.75–1.33)	0.992
*NINJ2*	rs75750647	Co-dominant	AA vs GG	70	57	1.22 (0.82–1.82)	0.321
			AG vs GG	210	221	0.94 (0.72–1.22)	0.632
		Dominant	AA-AG vs GG	280	278	1.00 (0.78–1.28)	0.973
		Recessive	AA vs AG-GG	70	57	1.26 (0.87–1.84)	0.224
		Additive	—	—	—	1.05 (0.88–1.26)	0.572
*NINJ2*	rs7307242	Co-dominant	AA vs TT	9	7	1.13 (0.42–3.09)	0.080
			AT vs TT	110	118	0.92 (0.68–1.24)	0.595
		Dominant	AA-AT vs TT	119	125	0.93 (0.70–1.25)	0.650
		Recessive	AA vs AT-TT	9	7	1.15 (0.42–3.14)	0.780
		Additive	—	—	—	0.96 (0.73–1.24)	0.736
*NINJ2*	rs10849390	Co-dominant	GG vs AA	76	59	1.27 (0.86–1.88)	0.231
			GA vs AA	208	217	0.93 (0.71–1.21)	0.579
		Dominant	GG-GA vs AA	284	276	1.00 (0.78–1.29)	0.997
		Recessive	GG vs GA-AA	76	59	1.32 (0.91–1.91)	0.140
		Additive	—	—	—	1.07 (0.89–1.28)	0.466
*NINJ2*	rs11610368	Co-dominant	AA vs GG	7	8	0.84 (0.30–2.38)	0.745
			AG vs GG	104	108	0.91 (0.67–1.24)	0.560
		Dominant	AA-AG vs GG	111	116	0.91 (0.67–1.22)	0.527
		Recessive	AA vs AG-GG	7	8	0.86 (0.30–2.42)	0.773
		Additive	—	—	—	0.91 (0.70–1.20)	0.516

SNP, single-nucleotide polymorphism; OR, odds ratio; 95% CI, 95% confidence interval. *p*-Value was calculated by logistic regression analysis with adjustment for age and gender. Bold data means significant difference (*p* < 0.05).

**Table 4:** Results of FPRP analysis for significant findings

Model	OR (95% CI)	Power	Prior probability				
			0.25	0.1	0.01	0.001	0.0001
rs16901963							
TA vs AA	0.67 (0.50–0.89)	0.978	0.017*	0.050*	0.366	0.853	0.983
TT vs TA-AA	1.55 (1.14–2.12)	0.945	0.019*	0.055*	0.390	0.866	0.985

^*^The level of FPRP threshold was set at 0.2 and noteworthy findings are presented.

### 3.3 Stratified analysis of the effect of MRPS30-DT and NINJ2 variants on lung cancer risk

We further performed the stratified analyses by age, gender, smoking, and alcohol consumption to explore the effect of MRPS30-DT and NINJ2 variants on lung cancer risk (Table 5). According to the gender-stratified analysis, heterozygote TA of MRPS30-DT rs16901963 was associated with lung cancer risk in males (heterozygote: adjusted OR = 0.66, 95% CI: 0.47–0.92, *p* = 0.013; recessive: adjusted OR = 1.48, 95% CI: 1.04–2.13, *p* = 0.032).

**Table 5:** Stratified analysis of the association of MRPS30-DT and NINJ2 polymorphisms with lung cancer risk

**SNP**	**Subgroups**	**Allele**		**Homozygote**		**Heterozygote**		**Dominant**		**Recessive**		**Additive**	
		**OR (95%CI)**	***p***-Value	**OR (95% CI)**	***p***-Value	**OR (95% CI)**	***p***-Value	**OR (95% CI)**	***p***-Value	**OR (95% CI)**	***p***-Value	**OR (95% CI)**	***p***-Value
***MRPS30-DT***													
rs16901963	Age (>59)	1.15 (0.90–1.48)	0.256	1.47 (0.91–2.38)	0.116	0.64 (0.43–0.96)	**0.030**	0.85 (0.58–1.23)	0.382	1.90 (1.25–2.91)	**0.003**	1.15 (0.91–1.46)	0.249
	Male	1.02 (0.83–1.25)	0.853	1.18 (0.79–1.77)	0.421	0.66 (0.47–0.92)	**0.013**	0.80 (0.59–1.08)	0.144	1.48 (1.04–2.13)	**0.032**	1.03 (0.84–1.25)	0.808
	Smoking	1.09 (0.80–1.48)	0.578	1.46 (0.76–2.80)	0.255	0.63 (0.39–1.04)	0.069	0.82 (0.51–1.29)	0.385	1.89 (1.06–3.39)	**0.032**	1.10 (0.82–1.48)	0.517
***NINJ2***													
rs75750647	Age (≤59)	1.31 (1.00–1.72)	0.049	1.95 (1.04–3.65)	**0.036**	1.14 (0.77–1.68)	0.515	1.28 (0.88–1.84)	0.195	1.84 (1.01–3.34)	**0.047**	1.30 (0.99–1.71)	0.058
rs10849390	Age (≤59)	1.09 (0.84–1.42)	0.524	1.64 (0.90–2.97)	0.105	0.82 (0.55–1.22)	0.324	0.96 (0.66–1.40)	0.832	1.81 (1.03–3.18)	**0.039**	1.13 (0.86–1.48)	0.380
rs11610368	Age (>59)	0.67 (0.45–0.99)	**0.043**	0.32 (0.06–1.58)	0.163	0.73 (0.47–1.14)	0.165	0.69 (0.45–1.06)	0.091	0.34 (0.07–1.68)	0.187	0.69 (0.47–1.02)	0.059
	Non-smoking	0.54 (0.32–0.90)	**0.018**	0.25 (0.03–2.50)	0.239	0.53 (0.28–0.98)	**0.042**	0.50 (0.27–0.92)	**0.025**	0.29 (0.03–2.81)	0.283	0.52 (0.30–0.91)	**0.021**
	Non-drinking	0.66 (0.44–1.00)	0.048	0.62 (0.16–2.46)	0.499	0.57 (0.35–0.94)	**0.026**	0.58 (0.36–0.93)	**0.023**	0.70 (0.18–2.75)	0.612	0.64 (0.42–0.96)	**0.033**

SNP, single-nucleotide polymorphism; OR, odds ratio; 95% CI, 95% confidence interval. *p*-Value was calculated by logistic regression analysis with adjustment for age and gender. Bold data means significant difference (*p* < 0.05).

We also selected the average age of 59 as the critical point for stratified analysis. Among patients aged ≤59 years, rs75750647 of NINJ2 was considered a risk factor for lung cancer in the homozygote (adjusted OR = 1.95, 95% CI: 1.04–3.65, *p* = 0.036) and recessive (adjusted OR = 1.84, 95% CI: 1.01–3.34, *p* = 0.047) models, while rs10849390 GG carriers had a 0.81-fold increased risk of lung cancer in the recessive model (95% CI: 1.03–3.18, *p* = 0.039). In patients over 59 years, rs11610368 A and rs16901963 TA were correlated with a decreased risk of lung cancer (adjusted OR = 0.67, 95% CI: 0.45–0.99, *p* = 0.043; adjusted OR = 0.64, 95% CI: 0.43–0.96, *p* = 0.030).

Besides, we conducted smoking- and drinking-stratified analysis to explore the association between MRPS30-DT and NINJ2 polymorphisms and the risk of lung cancer. MRPS30-DT rs16901963 was related to an increased risk of lung cancer in the recessive model (adjusted OR = 1.89, 95% CI: 1.06–3.39, *p* = 0.032) in smokers. However, in non-smoking and non-drinking patients, rs11610368 of NINJ2 was found to be significantly correlated with a reduced susceptibility to lung cancer under the allelic (*p* = 0.018 and *p* = 0.048, respectively), heterozygote (*p* = 0.042 and *p* = 0.026, respectively), dominant (*p* = 0.025 and *p* = 0.023, respectively), and additive (*p* = 0.021 and *p* = 0.033, respectively) models.

### 3.4 LD and haplotype analysis

In the results of LD analysis, we did not find linkage between MRPS30-DT (rs16901963 and rs2118763) and NINJ2 (rs118050317, rs75750647, rs7307242, rs10849390, and rs11610368). No haplotype was found to be associated with the risk of lung cancer (Table 6).

**Table 6:** Haplotype analysis of the association between MRPS30-DT and NINJ2 polymorphisms and lung cancer risk

**Gene**	**SNP**	**Haplotype**	**Fre-case**	**Fre-control**	***p*** ^**a**^	**OR (95% CI)**	***p*** ^**b**^
*MRPS30-DT*	rs16901963|rs2118763	AT	0.059	0.060	0.902	0.94 (0.65–1.37)	0.747
		TC	0.437	0.428	0.670	1.05 (0.89–1.25)	0.540
		AC	0.496	0.488	0.716	1.04 (0.88–1.24)	0.639
*NINJ2*	rs75750647|rs7307242	GA	0.345	0.334	0.607	1.06 (0.88–1.27)	0.537
		CG	0.107	0.104	0.843	1.00 (0.75–1.33)	0.989
		GG	0.453	0.439	0.540	1.05 (0.89–1.25)	0.553
*NINJ2*	rs10849390|rs11610368	GA	0.115	0.119	0.737	0.93 (0.70–1.23)	0.621
		GG	0.244	0.220	0.213	1.14 (0.93–1.40)	0.206
		AG	0.362	0.344	0.410	1.07 (0.90–1.29)	0.444

SNP, single-nucleotide polymorphism; OR, odds ratio; 95% CI, 95% confidence interval; Fre, frequency. ^a^ *p*-value was calculated by chi-square test. ^b^ *p*-value was calculated by logistic regression analysis with adjustment for age and gender. *p* < 0.05 indicates statistical significance.

### 3.5 SNP–SNP interactions and lung cancer risk.

**Figure 1:**
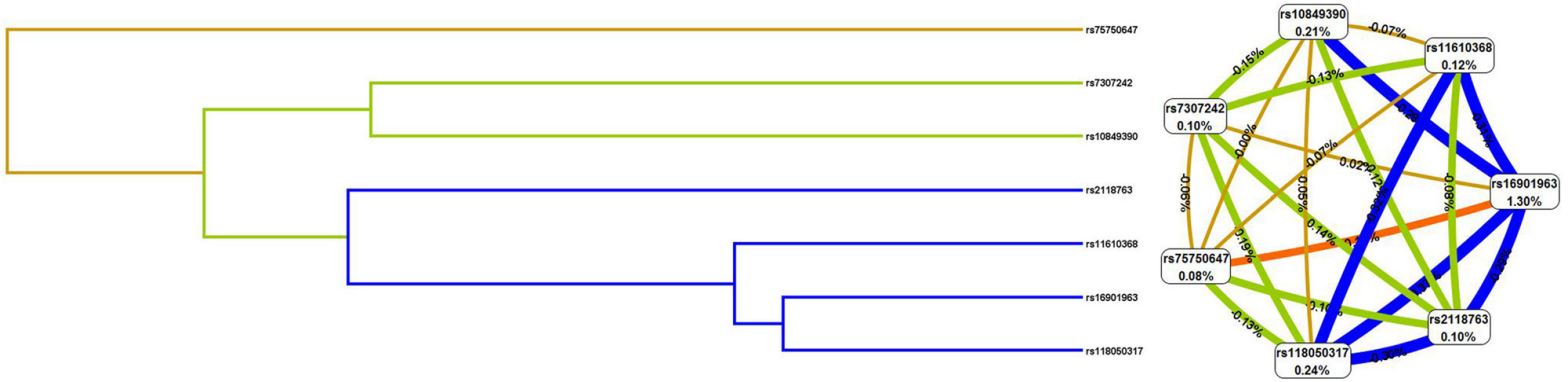
Potential SNP–SNP interactions for predicting the risk of lung cancer risk by MDR analysis.

In Table 7, MDR analysis showed that the model consisting of four loci (rs16901963, rs2118763, rs7307242, and rs11610368) was considered the best model, with the training accuracy of 56.5%, the testing accuracy of 55.7%, and the cross-validation consistency of 10/10. In Figure 1, the dendrogram (left) and the circle graph (right) indicated that rs16901963 and rs75750647 might play a synergistic role in predicting lung cancer risk.

**Table 7:** MDR analysis of SNP–SNP interactions of MRPS30-DT and NINJ2 variants

Model	Training Bal. Acc.	Testing Bal. Acc.	CVC	OR (95% CI)	*p-*Value
rs16901963	0.547	0.530	10/10	1.52 (1.12–2.07)	**0.007**
rs16901963, rs2118763	0.556	0.537	10/10	1.65 (1.29–2.12)	**<0.000**
rs16901963, rs75750647, rs7307242	0.562	0.540	10/10	2.18 (1.62–2.96)	**<0.000**
rs16901963, rs2118763, rs7307242, rs11610368	0.565	0.557	10/10	2.11 (1.58–2.83)	**<0.000**
rs16901963, rs118050317, rs75750647, rs10849390, rs11610368	0.551	0.525	10/10	3.15 (2.08–4.77)	**<0.000**
rs16901963, rs2118763, rs118050317, rs75750647, rs10849390, rs11610368	0.558	0.531	10/10	3.77 (2.38–5.95)	**<0.000**
rs16901963, rs2118763, rs118050317, rs75750647, rs7307242, rs10849390, rs11610368	0.545	0.519	10/10	4.39 (2.54–7.59)	**<0.000**

MDR: multi-factor dimensionality reduction; Bal. Acc.: balanced accuracy; CVC: cross-validation consistency; OR: odds ratio; 95% CI: 95% confidence interval. Bold data means significant difference (*p* < 0.05).

## 4 Discussion

In the present study, we found that MRPS30-DT rs16901963 was related to lung cancer risk in people aged >59 years, males, and smokers. NINJ2 (rs75750647 and rs10849390) increased the risk of lung cancer in people aged ≤59 years, while NINJ2 rs11610368 was correlated with a reduced risk of lung cancer in people aged >59 years, non-drinkers, and non-smokers. The above results will provide a theoretical basis for elucidating the pathogenesis of lung cancer.

Recently, one research reported by Wu et al. has shown that MRPS30-DT was overexpressed in breast cancer through microarray analysis and MRPS30-DT knockdown could significantly inhibit the proliferation and invasion of breast cancer cells [7]. Meanwhile, Yang et al. have put forward that BRCAT54, also known as MRPS30-DT, could inhibit the proliferation of non-small-cell lung cancer [8]. Thus, MRPS30-DT played an important role in the development of cancers. To better study the specific mechanism of MRPS30-DT in specific diseases, researchers have recently adopted a case–control strategy to further explore the possible role of MRPS30-DT polymorphisms in disease progression. At present, Chen et al. have found the contribution of the MRPS30-DT genetic polymorphisms to IgA nephropathy in the Chinese Han population [20]. However, the contribution of the MRPS30-DT genetic polymorphisms to lung cancer has not been discovered in the Chinese Han population. We are the first to report the relationship between MRPS30-DT rs16901963 and lung cancer risk in the Han population in people aged >59 years, males, and smokers. The results still need to be confirmed by subsequent experiments.

NINJ2 has also been observed by Li et al. and Zhou et al. to play a carcinogenic role in colorectal cancer and glioma [12,13]. Cheng et al. have investigated the relationship between NINJ2 variants (rs118050317, rs75750647, rs7307242, rs10849390, and rs11610368) and endometrial cancer susceptibility [21], showing that the rs118050317 mutant allele C was associated with an increased risk of endometrial cancer. In this case–control study, the relationship between rs118050317 and the risk of lung cancer was not found. Nevertheless, NINJ2 (rs75750647 and rs10849390) increased the risk of lung cancer in people aged ≤59 years, while NINJ2 rs11610368 was correlated with a reduced risk of lung cancer in people aged >59 years. Although this relationship was not found in drinkers and smokers, it would still be of great significance to explore the contribution of smoking and alcohol consumption to this correlation. Admittedly, this study has certain limitations. First of all, study subjects were all Han Chinese, and thus, it is necessary to validate our results in different ethnic populations in later studies. In addition, the influence of gene–environment interactions on lung cancer was less investigated due to the insufficient information about participants. Third, the sample size in this study was not large enough to stratify all subtypes of lung cancer. In the following studies, a larger sample size is needed to verify the current results, and related experiments will be conducted to explore the underlying molecular mechanisms of MRPS30-DT and NINJ2 in the regulation of lung cancer.

## 5 Conclusion

To conclude, we observed the relationship between genetic polymorphisms of MRPS30-DT and NINJ2 and the risk of lung cancer and proved that the effect of variants on lung cancer susceptibility was dependent on age, gender, smoking, and drinking status. Our findings provided a theoretical support for revealing the mechanisms of lung cancer.

## Appendix

**Table A1:** Information about primers in this study

SNP	1st-PCRP (5'-3')	2nd-PCRP (5′−3′)	UEP_DIR	UEP (5′−3′)
rs16901963	ACGTTGGATGACACTGGATTCACACACTGC	ACGTTGGATGTTGAGGACCGCAAAGCATAG	R	TGTAAATACCATTTCATGAAAAG
rs2118763	ACGTTGGATGCAGTATGCTAATGTAAAGGTG	ACGTTGGATGAGCCCAAAGAAAGCCAATTT	F	ccccTATGTGACAATGTTTCCTTTA
rs118050317	ACGTTGGATGACAGGAGCTGGTCATGTTGC	ACGTTGGATGGTGTAGTGATTGACACCTG	R	ggggtGCCGATGGGGAAGGATTAG
rs75750647	ACGTTGGATGCCCCCACAAAATTACAAACC	ACGTTGGATGTGTTCGCTGTGTACTGGATG	R	CTAAAGCAGGGTGGAG
rs7307242	ACGTTGGATGGCCCTAGCCTGTTTCTTTAG	ACGTTGGATGAAATGCTTCTCCTGGAAGTC	F	CCAGATCACTAGCTCTGA
rs10849390	ACGTTGGATGGAAATCAGTACTGCCTGTGC	ACGTTGGATGTCACACAATCTCACAGGGAC	F	tcgccCAGGGACAGCCCGCTGCC
rs11610368	ACGTTGGATGCTCTGCAATGTTACACAGCC	ACGTTGGATGTCTGTGACTCCTTGCCAATG	R	CCTTGCCAATGGATAGAATAGAA

SNP, single nucleotide polymorphism; PCRP, polymerase chain reaction primer; UEP_DIR, unique base extension primer direction; F, forward; R, reverse; UEP, unique base extension primer.
